# Ethyl 1-(4-methyl­phen­yl)-5-phenyl-4-phenyl­sulfon­yl-1*H*-pyrazole-3-carboxyl­ate

**DOI:** 10.1107/S1600536811036580

**Published:** 2011-09-14

**Authors:** Hatem A. Abdel-Aziz, Khalid A. Al-Rashood, Seik Weng Ng, Edward R. T. Tiekink

**Affiliations:** a Department of Pharmaceutical Chemistry, College of Pharmacy, King Saud University, Riyadh 11451, Saudi Arabia; b Department of Chemistry, University of Malaya, 50603 Kuala Lumpur, Malaysia; c Chemistry Department, Faculty of, Science, King Abdulaziz University, PO Box 80203 Jeddah, Saudi Arabia

## Abstract

The title compound, C_25_H_22_N_2_O_4_S, features a tetra-substituted pyrazole ring. The dihedral angles formed between the five-membered ring (r.m.s. deviation = 0.007 Å) and the N- and C-bound phenyl rings are 48.10 (7) and 72.01 (7) °, respectively, indicating that the planes through the residues are significantly twisted from the plane through the heterocycle. The ester-CO_2_ group is also twisted out of this plane, with an O—C—C—N torsion angle of −29.04 (11)°. The sulfonyl-O atoms lie to one side of the pyrazole plane and the sulfonyl­phenyl ring to the other. The dihedral angle between the two ring planes is 70.63 (7) °. Supra­molecular arrays are formed in the crystal structure sustained by C—H⋯O and C—H⋯π(pyrazole) inter­actions and methyl-C—H⋯π(N-bound benzene) contacts.

## Related literature

For background to the chemistry and biological activity of pyrazole derivatives, see: Abdel-Wahab *et al.* (2009 ▶); Abdel-Aziz *et al.* (2009 ▶, 2010 ▶).
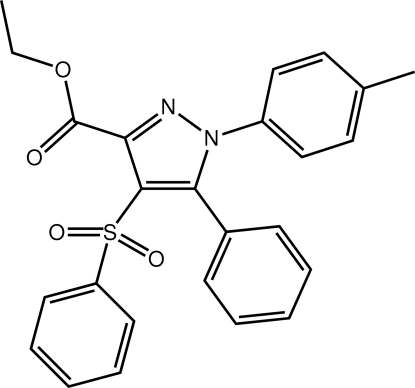


## Experimental

### 

#### Crystal data

C_25_H_22_N_2_O_4_S
*M* *_r_* = 446.51
Triclinic, 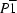

*a* = 7.2440 (3) Å
*b* = 11.0798 (5) Å
*c* = 14.8247 (5) Å
α = 68.818 (4)°
β = 87.773 (3)°
γ = 81.241 (4)°
*V* = 1096.36 (8) Å^3^
*Z* = 2
Cu *K*α radiation
μ = 1.60 mm^−1^
*T* = 100 K
0.40 × 0.30 × 0.20 mm

#### Data collection

Agilent SuperNova Dual diffractometer with Atlas detector
Absorption correction: multi-scan (*CrysAlis PRO*; Agilent, 2010 ▶) *T*_min_ = 0.566, *T*_max_ = 0.740
7378 measured reflections
4304 independent reflections
4106 reflections with *I* > 2σ(*I*)
*R*_int_ = 0.014

#### Refinement

*R*[*F* ^2^ > 2σ(*F* ^2^)] = 0.034
*wR*(*F* ^2^) = 0.091
*S* = 0.85
4304 reflections
290 parameters
H-atom parameters constrained
Δρ_max_ = 0.35 e Å^−3^
Δρ_min_ = −0.42 e Å^−3^

### 

Data collection: *CrysAlis PRO* (Agilent, 2010 ▶); cell refinement: *CrysAlis PRO*; data reduction: *CrysAlis PRO*; program(s) used to solve structure: *SHELXS97* (Sheldrick, 2008 ▶); program(s) used to refine structure: *SHELXL97* (Sheldrick, 2008 ▶); molecular graphics: *ORTEP-3* (Farrugia, 1997 ▶) and *DIAMOND* (Brandenburg, 2006 ▶); software used to prepare material for publication: *publCIF* (Westrip, 2010 ▶).

## Supplementary Material

Crystal structure: contains datablock(s) global, I. DOI: 10.1107/S1600536811036580/bt5639sup1.cif

Structure factors: contains datablock(s) I. DOI: 10.1107/S1600536811036580/bt5639Isup2.hkl

Supplementary material file. DOI: 10.1107/S1600536811036580/bt5639Isup3.cml

Additional supplementary materials:  crystallographic information; 3D view; checkCIF report

## Figures and Tables

**Table 1:** Hydrogen-bond geometry (Å, °) *Cg*1 and *Cg*2 are the centroids of the N1,N2,C4–C6 and C19–C24 rings, respectively.

*D*—H⋯*A*	*D*—H	H⋯*A*	*D*⋯*A*	*D*—H⋯*A*
C9—H9⋯O1^i^	0.95	2.45	3.2392 (19)	140
C16—H16⋯O2^ii^	0.95	2.49	3.3928 (18)	158
C17—H17⋯O1^iii^	0.95	2.50	3.3895 (18)	157
C18—H18⋯O2^iii^	0.95	2.58	3.4031 (17)	145
C23—H23⋯O4^iv^	0.95	2.59	3.3155 (18)	133
C15—H15⋯*Cg*1^i^	0.95	2.80	3.6781 (15)	154
C25—H25c⋯*Cg*2^v^	0.98	2.64	3.5649 (16)	157

Symmetry codes: (i) 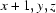; (ii) 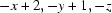; (iii) 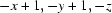; (iv) 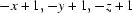; (v) 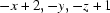.

**Fig. 1.**
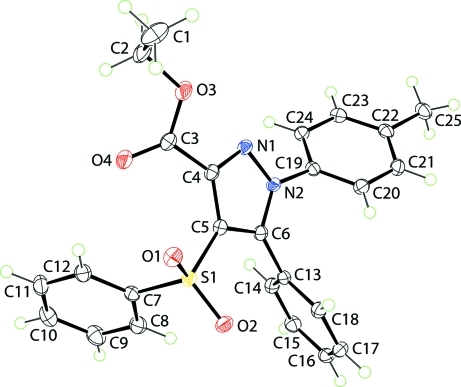
The molecular structure of (I) showing the atom-labelling scheme and displacement ellipsoids at the 50% probability level.

**Fig. 2.**
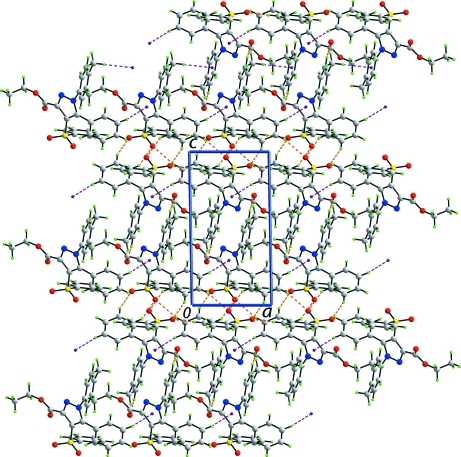
Supramolecular array in the ac plane in (I) mediated by C—H···O and C—H···π interactions shown as orange and purple dashed lines, respectively.

**Fig. 3.**
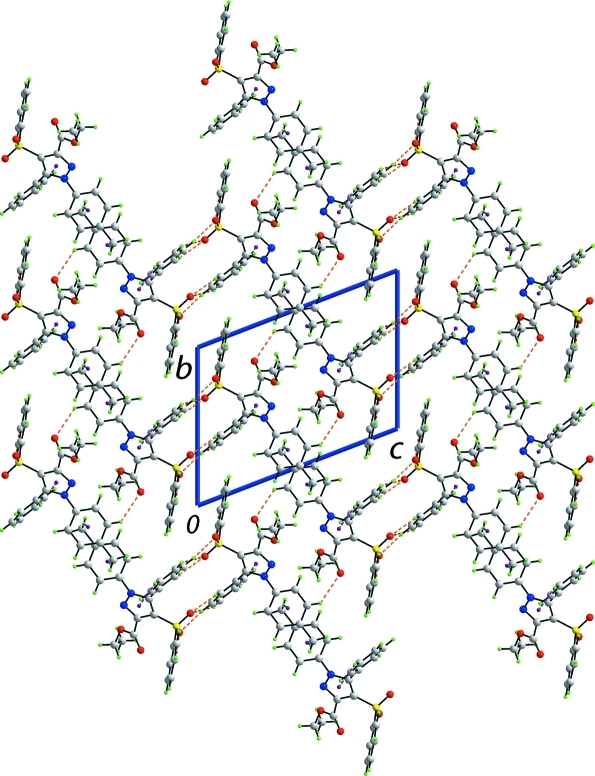
A view in projection down the a axis of the unit-cell contents of (I). The C—H···O and C—H···π interactions are shown as orange and purple dashed lines, respectively.

## supplementary crystallographic information

### Comment

Our previous work evaluating the biological potential of pyrazole derivatives (Abdel-Wahab *et al.*, 2009; Abdel-Aziz *et al.*, 2009; Abdel-Aziz *et al.*, 2010) lead to the characterization of the title compound, (I).

The molecular structure of (I), Fig. 1, features a tetra-substituted pyrazole ring. The ester group is twisted out of the plane through the five-membered ring (r.m.s. deviation = 0.007 Å) as seen in the value of the O3—C3—C4—N1 torsion angle of -29.04 (11) °. The ring-connected benzene rings, (C13–C18) and (19–C24), form dihedral angles of 72.01 (7) and 48.10 (7) °, respectively, with the pyrazole ring also indicating significant twists; the dihedral angle between the two ring-bound benzene rings is 71.93 (7) °. With respect to the least-squares plane through the pyrazole ring, the sulfonyl-O atoms lie to one side, and the benzene ring to the other; the dihedral angle between the pyrazole and sulfonyl-benzene rings is 70.63 (7) °, indicating an almost orthogonal relationship.

The crystal structure features supramolecular arrays in the *ac* plane sustained by C—H···O and C—H···π interactions [involving the pyrazole ring as the acceptor], Fig. 2 and Table 1. Layers are connected along the *b* direction by C—H···.π interactions involving methyl-H and the N-bound benzene ring, Fig. 3 and Table 1.

### Experimental

1-Phenyl-2-(phenylsulfonyl)ethanone (0.26 g, 1 mmol) was added to a stirred ethanolic sodium ethoxide solution [prepared from sodium metal (0.023 g, 1 mmol) and 25 ml of absolute ethanol]. After stirring for 20 min, ethyl 2-chloro-2-(2-*p*-tolylhydrazono)acetate (0.241 g, 1 mmol) was added and the reaction mixture was left to stir at room temperature for 12 h. Cold water (50 ml) was then added. The solid product was collected by filtration, washed with water and dried. Recrystallization from ethanol afforded the title pyrazole. The yellow blocks were isolated from its ethanol solution by slow evaporation at room temperature

### Refinement

H-atoms were placed in calculated positions [C—H 0.95 to 0.99 Å, *U*_iso_(H) 1.2 to 1.5*U*_eq_(C)] and were included in the refinement in the riding model approximation.

### Crystal data

**Table:**

C_25_H_22_N_2_O_4_S	*Z* = 2
*M**_r_* = 446.51	*F*(000) = 468
Triclinic, *P*1	*D*_x_ = 1.353 Mg m^−^^3^
Hall symbol: -P 1	Cu *K*α radiation, λ = 1.54184 Å
*a* = 7.2440 (3) Å	Cell parameters from 5331 reflections
*b* = 11.0798 (5) Å	θ = 3.2–74.1°
*c* = 14.8247 (5) Å	µ = 1.60 mm^−^^1^
α = 68.818 (4)°	*T* = 100 K
β = 87.773 (3)°	Block, yellow
γ = 81.241 (4)°	0.40 × 0.30 × 0.20 mm
*V* = 1096.36 (8) Å^3^	

### Data collection

**Table:**

Agilent SuperNova Dual diffractometer with Atlas detector	4304 independent reflections
Radiation source: SuperNova (Cu) X-ray Source	4106 reflections with *I* > 2σ(*I*)
mirror	*R*_int_ = 0.014
Detector resolution: 10.4041 pixels mm^-1^	θ_max_ = 74.3°, θ_min_ = 3.2°
ω scan	*h* = −8→8
Absorption correction: multi-scan (*CrysAlis PRO*; Agilent, 2010)	*k* = −12→13
*T*_min_ = 0.566, *T*_max_ = 0.740	*l* = −18→15
7378 measured reflections	

### Refinement

**Table:**

Refinement on *F*^2^	Primary atom site location: structure-invariant direct methods
Least-squares matrix: full	Secondary atom site location: difference Fourier map
*R*[*F*^2^ > 2σ(*F*^2^)] = 0.034	Hydrogen site location: inferred from neighbouring sites
*wR*(*F*^2^) = 0.091	H-atom parameters constrained
*S* = 0.85	*w* = 1/[σ^2^(*F*_o_^2^) + (0.060*P*)^2^ + 0.8665*P*] where *P* = (*F*_o_^2^ + 2*F*_c_^2^)/3
4304 reflections	(Δ/σ)_max_ < 0.001
290 parameters	Δρ_max_ = 0.35 e Å^−^^3^
0 restraints	Δρ_min_ = −0.42 e Å^−^^3^

### Special details

**Table:**

Geometry. All e.s.d.'s (except the e.s.d. in the dihedral angle between two l.s. planes) are estimated using the full covariance matrix. The cell e.s.d.'s are taken into account individually in the estimation of e.s.d.'s in distances, angles and torsion angles; correlations between e.s.d.'s in cell parameters are only used when they are defined by crystal symmetry. An approximate (isotropic) treatment of cell e.s.d.'s is used for estimating e.s.d.'s involving l.s. planes.
Refinement. Refinement of *F*^2^ against ALL reflections. The weighted *R*-factor *wR* and goodness of fit *S* are based on *F*^2^, conventional *R*-factors *R* are based on *F*, with *F* set to zero for negative *F*^2^. The threshold expression of *F*^2^ > σ(*F*^2^) is used only for calculating *R*-factors(gt) *etc*. and is not relevant to the choice of reflections for refinement. *R*-factors based on *F*^2^ are statistically about twice as large as those based on *F*, and *R*- factors based on ALL data will be even larger.

### Fractional atomic coordinates and isotropic or equivalent isotropic displacement parameters (Å^2^)

**Table:**

	*x*	*y*	*z*	*U*_iso_*/*U*_eq_	
S1	0.44173 (4)	0.70984 (3)	0.10567 (2)	0.01551 (10)	
O1	0.24561 (13)	0.75817 (9)	0.09106 (7)	0.0201 (2)	
O2	0.54033 (14)	0.66162 (9)	0.03658 (7)	0.0202 (2)	
O3	0.08177 (14)	0.59487 (10)	0.38398 (9)	0.0275 (2)	
O4	0.19467 (16)	0.77438 (10)	0.28475 (8)	0.0280 (2)	
N1	0.40421 (15)	0.44865 (11)	0.36938 (8)	0.0161 (2)	
N2	0.55383 (15)	0.39169 (10)	0.33340 (8)	0.0150 (2)	
C1	−0.2495 (2)	0.66923 (19)	0.37793 (12)	0.0333 (4)	
H1A	−0.3481	0.7238	0.3990	0.050*	
H1B	−0.2743	0.5785	0.4023	0.050*	
H1C	−0.2470	0.7016	0.3071	0.050*	
C2	−0.0656 (2)	0.67488 (16)	0.41664 (13)	0.0286 (3)	
H2A	−0.0413	0.7666	0.3935	0.034*	
H2B	−0.0672	0.6418	0.4882	0.034*	
C3	0.20136 (18)	0.65804 (13)	0.31954 (9)	0.0171 (3)	
C4	0.35304 (18)	0.56389 (12)	0.30001 (9)	0.0156 (3)	
C5	0.46821 (18)	0.58099 (12)	0.21819 (9)	0.0152 (3)	
C6	0.59874 (18)	0.46782 (12)	0.24248 (9)	0.0148 (3)	
C7	0.55863 (19)	0.83215 (12)	0.11696 (9)	0.0171 (3)	
C8	0.7518 (2)	0.82100 (14)	0.10621 (10)	0.0225 (3)	
H8	0.8189	0.7490	0.0927	0.027*	
C9	0.8443 (2)	0.91673 (15)	0.11553 (12)	0.0272 (3)	
H9	0.9760	0.9106	0.1086	0.033*	
C10	0.7444 (2)	1.02163 (14)	0.13504 (11)	0.0271 (3)	
H10	0.8083	1.0868	0.1417	0.033*	
C11	0.5522 (2)	1.03192 (14)	0.14486 (11)	0.0247 (3)	
H11	0.4851	1.1045	0.1577	0.030*	
C12	0.4571 (2)	0.93691 (13)	0.13606 (10)	0.0206 (3)	
H12	0.3254	0.9434	0.1429	0.025*	
C13	0.76709 (18)	0.42800 (12)	0.19410 (9)	0.0149 (3)	
C14	0.94252 (19)	0.42706 (13)	0.23053 (10)	0.0182 (3)	
H14	0.9520	0.4530	0.2845	0.022*	
C15	1.10313 (19)	0.38814 (13)	0.18770 (10)	0.0207 (3)	
H15	1.2224	0.3885	0.2120	0.025*	
C16	1.0902 (2)	0.34872 (13)	0.10965 (10)	0.0209 (3)	
H16	1.2004	0.3216	0.0808	0.025*	
C17	0.9159 (2)	0.34899 (13)	0.07374 (10)	0.0204 (3)	
H17	0.9071	0.3216	0.0205	0.025*	
C18	0.75398 (19)	0.38910 (13)	0.11527 (9)	0.0179 (3)	
H18	0.6350	0.3900	0.0901	0.022*	
C19	0.63396 (17)	0.26085 (12)	0.39171 (9)	0.0155 (3)	
C20	0.66215 (19)	0.16297 (13)	0.35283 (10)	0.0184 (3)	
H20	0.6400	0.1831	0.2859	0.022*	
C21	0.72330 (19)	0.03502 (13)	0.41333 (10)	0.0203 (3)	
H21	0.7447	−0.0321	0.3869	0.024*	
C22	0.75393 (19)	0.00307 (13)	0.51205 (10)	0.0185 (3)	
C23	0.72672 (19)	0.10390 (13)	0.54866 (10)	0.0188 (3)	
H23	0.7490	0.0843	0.6155	0.023*	
C24	0.66765 (18)	0.23258 (13)	0.48902 (10)	0.0173 (3)	
H24	0.6506	0.3005	0.5148	0.021*	
C25	0.8151 (2)	−0.13619 (13)	0.57784 (11)	0.0236 (3)	
H25A	0.7437	−0.1943	0.5618	0.035*	
H25B	0.7929	−0.1440	0.6452	0.035*	
H25C	0.9485	−0.1610	0.5694	0.035*	

### Atomic displacement parameters (Å^2^)

**Table:**

	*U*^11^	*U*^22^	*U*^33^	*U*^12^	*U*^13^	*U*^23^
S1	0.01730 (17)	0.01487 (16)	0.01383 (16)	−0.00005 (12)	−0.00058 (11)	−0.00538 (12)
O1	0.0178 (5)	0.0213 (5)	0.0204 (5)	0.0010 (4)	−0.0032 (4)	−0.0078 (4)
O2	0.0245 (5)	0.0202 (5)	0.0160 (5)	0.0000 (4)	0.0020 (4)	−0.0081 (4)
O3	0.0186 (5)	0.0235 (5)	0.0454 (7)	−0.0040 (4)	0.0133 (4)	−0.0193 (5)
O4	0.0408 (6)	0.0168 (5)	0.0235 (5)	0.0020 (4)	0.0076 (4)	−0.0071 (4)
N1	0.0138 (5)	0.0168 (5)	0.0186 (5)	−0.0007 (4)	0.0022 (4)	−0.0082 (4)
N2	0.0137 (5)	0.0147 (5)	0.0161 (5)	0.0000 (4)	0.0019 (4)	−0.0059 (4)
C1	0.0208 (8)	0.0498 (10)	0.0336 (9)	0.0035 (7)	0.0001 (6)	−0.0237 (8)
C2	0.0190 (7)	0.0290 (8)	0.0450 (9)	−0.0018 (6)	0.0104 (6)	−0.0237 (7)
C3	0.0162 (6)	0.0197 (6)	0.0172 (6)	−0.0001 (5)	−0.0026 (5)	−0.0096 (5)
C4	0.0145 (6)	0.0162 (6)	0.0174 (6)	−0.0017 (5)	−0.0006 (5)	−0.0079 (5)
C5	0.0158 (6)	0.0146 (6)	0.0159 (6)	−0.0019 (5)	−0.0003 (5)	−0.0065 (5)
C6	0.0153 (6)	0.0156 (6)	0.0151 (6)	−0.0035 (5)	−0.0003 (5)	−0.0068 (5)
C7	0.0204 (7)	0.0145 (6)	0.0143 (6)	−0.0021 (5)	−0.0003 (5)	−0.0029 (5)
C8	0.0215 (7)	0.0195 (7)	0.0240 (7)	−0.0006 (5)	0.0022 (5)	−0.0062 (6)
C9	0.0211 (7)	0.0243 (7)	0.0330 (8)	−0.0051 (6)	0.0006 (6)	−0.0059 (6)
C10	0.0314 (8)	0.0199 (7)	0.0294 (8)	−0.0087 (6)	−0.0025 (6)	−0.0059 (6)
C11	0.0310 (8)	0.0150 (6)	0.0271 (7)	−0.0011 (6)	−0.0006 (6)	−0.0072 (6)
C12	0.0212 (7)	0.0172 (6)	0.0208 (7)	0.0002 (5)	−0.0005 (5)	−0.0050 (5)
C13	0.0164 (6)	0.0118 (6)	0.0152 (6)	−0.0012 (5)	0.0020 (5)	−0.0037 (5)
C14	0.0192 (7)	0.0178 (6)	0.0193 (6)	−0.0036 (5)	0.0011 (5)	−0.0083 (5)
C15	0.0160 (7)	0.0202 (7)	0.0249 (7)	−0.0030 (5)	0.0014 (5)	−0.0070 (5)
C16	0.0209 (7)	0.0169 (6)	0.0223 (7)	−0.0007 (5)	0.0075 (5)	−0.0055 (5)
C17	0.0264 (7)	0.0179 (6)	0.0171 (6)	−0.0013 (5)	0.0033 (5)	−0.0075 (5)
C18	0.0195 (7)	0.0173 (6)	0.0173 (6)	−0.0020 (5)	−0.0006 (5)	−0.0068 (5)
C19	0.0122 (6)	0.0137 (6)	0.0188 (6)	−0.0014 (5)	0.0018 (5)	−0.0040 (5)
C20	0.0184 (7)	0.0191 (6)	0.0183 (6)	−0.0026 (5)	0.0015 (5)	−0.0077 (5)
C21	0.0200 (7)	0.0167 (6)	0.0258 (7)	−0.0025 (5)	0.0037 (5)	−0.0099 (5)
C22	0.0134 (6)	0.0166 (6)	0.0233 (7)	−0.0025 (5)	0.0029 (5)	−0.0046 (5)
C23	0.0169 (6)	0.0199 (7)	0.0177 (6)	−0.0028 (5)	0.0013 (5)	−0.0046 (5)
C24	0.0165 (6)	0.0168 (6)	0.0192 (6)	−0.0022 (5)	0.0020 (5)	−0.0073 (5)
C25	0.0216 (7)	0.0168 (7)	0.0278 (7)	−0.0017 (5)	0.0031 (6)	−0.0033 (6)

### Geometric parameters (Å, °)

**Table:**

S1—O1	1.4354 (10)	C11—C12	1.388 (2)
S1—O2	1.4378 (10)	C11—H11	0.9500
S1—C5	1.7544 (13)	C12—H12	0.9500
S1—C7	1.7624 (14)	C13—C18	1.3947 (18)
O3—C3	1.3380 (17)	C13—C14	1.3971 (19)
O3—C2	1.4635 (16)	C14—C15	1.3894 (19)
O4—C3	1.1968 (17)	C14—H14	0.9500
N1—C4	1.3274 (17)	C15—C16	1.387 (2)
N1—N2	1.3592 (15)	C15—H15	0.9500
N2—C6	1.3646 (17)	C16—C17	1.389 (2)
N2—C19	1.4362 (16)	C16—H16	0.9500
C1—C2	1.489 (2)	C17—C18	1.3909 (19)
C1—H1A	0.9800	C17—H17	0.9500
C1—H1B	0.9800	C18—H18	0.9500
C1—H1C	0.9800	C19—C24	1.3843 (19)
C2—H2A	0.9900	C19—C20	1.3875 (18)
C2—H2B	0.9900	C20—C21	1.3897 (19)
C3—C4	1.4913 (17)	C20—H20	0.9500
C4—C5	1.4165 (18)	C21—C22	1.394 (2)
C5—C6	1.3911 (18)	C21—H21	0.9500
C6—C13	1.4809 (17)	C22—C23	1.3942 (19)
C7—C12	1.3891 (19)	C22—C25	1.5054 (18)
C7—C8	1.394 (2)	C23—C24	1.3893 (19)
C8—C9	1.387 (2)	C23—H23	0.9500
C8—H8	0.9500	C24—H24	0.9500
C9—C10	1.389 (2)	C25—H25A	0.9800
C9—H9	0.9500	C25—H25B	0.9800
C10—C11	1.386 (2)	C25—H25C	0.9800
C10—H10	0.9500		
			
O1—S1—O2	119.33 (6)	C12—C11—H11	119.8
O1—S1—C5	106.92 (6)	C10—C11—H11	119.8
O2—S1—C5	106.93 (6)	C11—C12—C7	118.58 (13)
O1—S1—C7	109.11 (6)	C11—C12—H12	120.7
O2—S1—C7	107.81 (6)	C7—C12—H12	120.7
C5—S1—C7	105.98 (6)	C18—C13—C14	119.85 (12)
C3—O3—C2	117.09 (11)	C18—C13—C6	121.66 (12)
C4—N1—N2	104.77 (10)	C14—C13—C6	118.48 (11)
N1—N2—C6	113.13 (10)	C15—C14—C13	119.85 (12)
N1—N2—C19	117.27 (10)	C15—C14—H14	120.1
C6—N2—C19	129.49 (11)	C13—C14—H14	120.1
C2—C1—H1A	109.5	C16—C15—C14	120.34 (13)
C2—C1—H1B	109.5	C16—C15—H15	119.8
H1A—C1—H1B	109.5	C14—C15—H15	119.8
C2—C1—H1C	109.5	C15—C16—C17	119.82 (12)
H1A—C1—H1C	109.5	C15—C16—H16	120.1
H1B—C1—H1C	109.5	C17—C16—H16	120.1
O3—C2—C1	109.39 (12)	C16—C17—C18	120.42 (13)
O3—C2—H2A	109.8	C16—C17—H17	119.8
C1—C2—H2A	109.8	C18—C17—H17	119.8
O3—C2—H2B	109.8	C17—C18—C13	119.72 (13)
C1—C2—H2B	109.8	C17—C18—H18	120.1
H2A—C2—H2B	108.2	C13—C18—H18	120.1
O4—C3—O3	125.36 (12)	C24—C19—C20	120.89 (12)
O4—C3—C4	123.58 (13)	C24—C19—N2	118.50 (11)
O3—C3—C4	110.98 (11)	C20—C19—N2	120.40 (12)
N1—C4—C5	111.39 (11)	C21—C20—C19	119.00 (12)
N1—C4—C3	118.83 (11)	C21—C20—H20	120.5
C5—C4—C3	129.57 (12)	C19—C20—H20	120.5
C6—C5—C4	105.42 (11)	C20—C21—C22	121.40 (13)
C6—C5—S1	126.52 (10)	C20—C21—H21	119.3
C4—C5—S1	127.84 (10)	C22—C21—H21	119.3
N2—C6—C5	105.29 (11)	C23—C22—C21	118.19 (12)
N2—C6—C13	121.29 (11)	C23—C22—C25	120.56 (13)
C5—C6—C13	133.20 (12)	C21—C22—C25	121.25 (13)
C12—C7—C8	121.65 (13)	C24—C23—C22	121.16 (13)
C12—C7—S1	119.59 (11)	C24—C23—H23	119.4
C8—C7—S1	118.76 (10)	C22—C23—H23	119.4
C9—C8—C7	118.91 (13)	C19—C24—C23	119.33 (12)
C9—C8—H8	120.5	C19—C24—H24	120.3
C7—C8—H8	120.5	C23—C24—H24	120.3
C10—C9—C8	119.93 (14)	C22—C25—H25A	109.5
C10—C9—H9	120.0	C22—C25—H25B	109.5
C8—C9—H9	120.0	H25A—C25—H25B	109.5
C9—C10—C11	120.51 (14)	C22—C25—H25C	109.5
C9—C10—H10	119.7	H25A—C25—H25C	109.5
C11—C10—H10	119.7	H25B—C25—H25C	109.5
C12—C11—C10	120.40 (13)		
			
C4—N1—N2—C6	−0.25 (14)	C12—C7—C8—C9	0.4 (2)
C4—N1—N2—C19	−176.77 (11)	S1—C7—C8—C9	−179.68 (11)
C3—O3—C2—C1	110.72 (15)	C7—C8—C9—C10	−0.2 (2)
C2—O3—C3—O4	−2.0 (2)	C8—C9—C10—C11	−0.3 (2)
C2—O3—C3—C4	174.84 (12)	C9—C10—C11—C12	0.5 (2)
N2—N1—C4—C5	0.83 (14)	C10—C11—C12—C7	−0.2 (2)
N2—N1—C4—C3	−174.42 (11)	C8—C7—C12—C11	−0.3 (2)
O4—C3—C4—N1	147.76 (14)	S1—C7—C12—C11	179.87 (11)
O3—C3—C4—N1	−29.15 (17)	N2—C6—C13—C18	109.94 (15)
O4—C3—C4—C5	−26.5 (2)	C5—C6—C13—C18	−76.46 (19)
O3—C3—C4—C5	156.60 (13)	N2—C6—C13—C14	−68.59 (16)
N1—C4—C5—C6	−1.11 (15)	C5—C6—C13—C14	105.00 (17)
C3—C4—C5—C6	173.49 (13)	C18—C13—C14—C15	0.40 (19)
N1—C4—C5—S1	173.74 (10)	C6—C13—C14—C15	178.96 (12)
C3—C4—C5—S1	−11.7 (2)	C13—C14—C15—C16	−0.8 (2)
O1—S1—C5—C6	145.06 (11)	C14—C15—C16—C17	0.4 (2)
O2—S1—C5—C6	16.17 (13)	C15—C16—C17—C18	0.3 (2)
C7—S1—C5—C6	−98.65 (12)	C16—C17—C18—C13	−0.6 (2)
O1—S1—C5—C4	−28.76 (13)	C14—C13—C18—C17	0.31 (19)
O2—S1—C5—C4	−157.65 (12)	C6—C13—C18—C17	−178.21 (12)
C7—S1—C5—C4	87.53 (13)	N1—N2—C19—C24	−46.78 (16)
N1—N2—C6—C5	−0.43 (14)	C6—N2—C19—C24	137.37 (14)
C19—N2—C6—C5	175.56 (12)	N1—N2—C19—C20	127.96 (13)
N1—N2—C6—C13	174.73 (11)	C6—N2—C19—C20	−47.90 (19)
C19—N2—C6—C13	−9.3 (2)	C24—C19—C20—C21	0.6 (2)
C4—C5—C6—N2	0.88 (14)	N2—C19—C20—C21	−173.97 (12)
S1—C5—C6—N2	−174.06 (10)	C19—C20—C21—C22	1.0 (2)
C4—C5—C6—C13	−173.44 (13)	C20—C21—C22—C23	−1.8 (2)
S1—C5—C6—C13	11.6 (2)	C20—C21—C22—C25	178.30 (13)
O1—S1—C7—C12	16.54 (13)	C21—C22—C23—C24	1.1 (2)
O2—S1—C7—C12	147.52 (11)	C25—C22—C23—C24	−179.04 (12)
C5—S1—C7—C12	−98.27 (11)	C20—C19—C24—C23	−1.4 (2)
O1—S1—C7—C8	−163.34 (10)	N2—C19—C24—C23	173.35 (11)
O2—S1—C7—C8	−32.36 (12)	C22—C23—C24—C19	0.5 (2)
C5—S1—C7—C8	81.85 (12)		

### Hydrogen-bond geometry (Å, °)

**Table:**

Cg1 and Cg2 are the centroids of the N1,N2,C4–C6 and C19–C24 rings, respectively.

**Table:**

*D*—H···*A*	*D*—H	H···*A*	*D*···*A*	*D*—H···*A*
C9—H9···O1^i^	0.95	2.45	3.2392 (19)	140
C16—H16···O2^ii^	0.95	2.49	3.3928 (18)	158
C17—H17···O1^iii^	0.95	2.50	3.3895 (18)	157
C18—H18···O2^iii^	0.95	2.58	3.4031 (17)	145
C23—H23···O4^iv^	0.95	2.59	3.3155 (18)	133
C15—H15···Cg1^i^	0.95	2.80	3.6781 (15)	154
C25—H25c···Cg2^v^	0.98	2.64	3.5649 (16)	157

Symmetry codes: (i) *x*+1, *y*, *z*; (ii) −*x*+2, −*y*+1, −*z*; (iii) −*x*+1, −*y*+1, −*z*; (iv) −*x*+1, −*y*+1, −*z*+1; (v) −*x*+2, −*y*, −*z*+1.
